# Acute PDE4 Inhibition Induces a Transient Increase in Blood Glucose in Mice

**DOI:** 10.3390/ijms24043260

**Published:** 2023-02-07

**Authors:** Daniel Irelan, Abigail Boyd, Edward Fiedler, Peter Lochmaier, Will McDonough, Ileana V. Aragon, Lyudmila Rachek, Lina Abou Saleh, Wito Richter

**Affiliations:** 1 Department of Biochemistry & Molecular Biology and Center for Lung Biology, Whiddon College of Medicine, University of South Alabama, Mobile, AL 36688, USA; 2 Department of Pharmacology, Whiddon College of Medicine, University of South Alabama, Mobile, AL 36688, USA

**Keywords:** cAMP-phosphodiesterase, PDE4, blood glucose, adrenergic signaling, insulin, 2-deoxyglucose, skeletal muscle

## Abstract

cAMP-phosphodiesterase 4 (PDE4) inhibitors are currently approved for the treatment of inflammatory diseases. There is interest in expanding the therapeutic application of PDE4 inhibitors to metabolic disorders, as their chronic application induces weight loss in patients and animals and improves glucose handling in mouse models of obesity and diabetes. Unexpectedly, we have found that acute PDE4 inhibitor treatment induces a temporary increase, rather than a decrease, in blood glucose levels in mice. Blood glucose levels in postprandial mice increase rapidly upon drug injection, reaching a maximum after ~45 min, and returning to baseline within ~4 h. This transient blood glucose spike is replicated by several structurally distinct PDE4 inhibitors, suggesting that it is a class effect of PDE4 inhibitors. PDE4 inhibitor treatment does not reduce serum insulin levels, and the subsequent injection of insulin potently reduces PDE4 inhibitor-induced blood glucose levels, suggesting that the glycemic effects of PDE4 inhibition are independent of changes in insulin secretion and/or sensitivity. Conversely, PDE4 inhibitors induce a rapid reduction in skeletal muscle glycogen levels and potently inhibit the uptake of 2-deoxyglucose into muscle tissues. This suggests that reduced glucose uptake into muscle tissue is a significant contributor to the transient glycemic effects of PDE4 inhibitors in mice.

## 1. Introduction

The ubiquitous second messenger 3′,5′-cyclic adenosine monophosphate (cAMP) transduces the action of myriad extracellular signals into distinct cellular responses and, thus, affects many physiologic paradigms, ranging from memory formation and cardiac output to reproduction and immune responses. This also extends to various metabolic functions, including adipogenesis, lipolysis, mitochondrial biogenesis, and the control of blood glucose levels [1,2,3]. In the fed state, the liver, skeletal muscle, and adipose tissue are major sites for blood glucose disposal, while in fasted or starved states, the liver and, to a lesser extent, the kidneys and intestines release glucose back into the bloodstream to provide fuel for cells and tissues that are unable to utilize fatty acids (e.g., the brain and red blood cells) [4,5,6]. Intriguingly, an increase in cAMP levels within distinct cells or tissues does not necessarily alter blood glucose levels in the same direction but may essentially produce opposite effects. For example, an increase in cAMP levels in pancreatic β-cells promotes insulin secretion and, thus, a decrease in blood glucose levels [7]. Conversely, increased cAMP signaling upon the activation of G_s_-coupled glucagon receptors in hepatocytes promotes glycogenolysis and/or gluconeogenesis to increase hepatic glucose release, thus increasing blood glucose levels [8]. This dichotomy in the outcome of cAMP signaling in distinct cell types elegantly redirects the flow of glucose between tissues during the switch between fed and fasted states, or between periods of rest and activity (e.g., exercise or sympatho-adrenergic stimulation) [2,8]. Consequently, approaches to modulating cAMP signaling as a way to therapeutically alter blood glucose levels would need to be targeted in a cell- and tissue-specific fashion.

The cellular concentration of cAMP is determined by the equilibrium between the rate of its production and the rate of its degradation. A plethora of extracellular signals (hormones, neurotransmitters, etc.) activate transmembrane receptors that couple to G proteins that stimulate (G_s_) or inhibit (G_i_) adenylyl cyclase activity, thereby fine-tuning the rate of cellular cAMP synthesis. The hydrolysis and inactivation of cAMP is mediated by cyclic nucleotide phosphodiesterases (PDEs). In humans and most mammalian model species, PDEs comprise a large group of enzymes that are encoded by 21 genes and can be grouped into 11 PDE families by their sequence homology and kinetic and pharmacologic properties [9,10]. Some PDE families exclusively hydrolyze cAMP (PDE4, PDE7, and PDE8), whereas others hydrolyze cAMP as well as the related second messenger, cGMP (PDE1, PDE2, PDE3, PDE10, and PDE11). A third group of PDEs is selective for cGMP (PDE5, PDE6, and PDE9) and, hence, is not directly involved in the regulation of cAMP signaling.

The cAMP-specific PDE family 4 (PDE4) is the largest PDE family. It comprises four genes or subtypes, PDE4A, PDE4B, PDE4C, and PDE4D. Each is expressed as multiple protein variants, which, in turn, are widely distributed throughout the body [11,12]. Non-/PAN-selective inhibitors of PDE4 enzymes produce a range of potential therapeutic effects in animal models, including potent anti-inflammatory, memory- and cognition-enhancing, or cardiovascular effects, and several PAN-PDE4 inhibitors have already been approved for clinical use for chronic obstructive pulmonary disease (COPD) and psoriasis [13,14,15,16,17,18,19,20]. More recently, PDE4 has also been proposed as a therapeutic target in metabolic disorders, including as a weight-loss strategy for obesity [21,22,23,24,25,26,27]. Intriguingly, several studies have shown that chronic treatment with the PAN-PDE4 inhibitor roflumilast reduces blood glucose levels in mouse models of obesity, suggesting a therapeutic potential for targeting PDE4 in diabetes [28,29]. This effect of PDE4 inhibition is paralleled by, and is perhaps dependent upon, elevated mitochondrial biogenesis and a resulting increase in energy expenditure [28]. Thus, the blood glucose-lowering effect appears dependent upon PDE4 being inhibited for extended time periods. During experiments to further delineate the role of PDE4 in regulating blood glucose levels, we unexpectedly observed that acute treatment with the PDE4 inhibitor roflumilast increased, rather than decreased, blood glucose levels in mice, and have further explored this observation.

## 2. Results

### 2.1. Treatment with the PDE4 Inhibitor Roflumilast Produces a Transient Increase in Blood Glucose Levels in Mice

**Figure 1:**
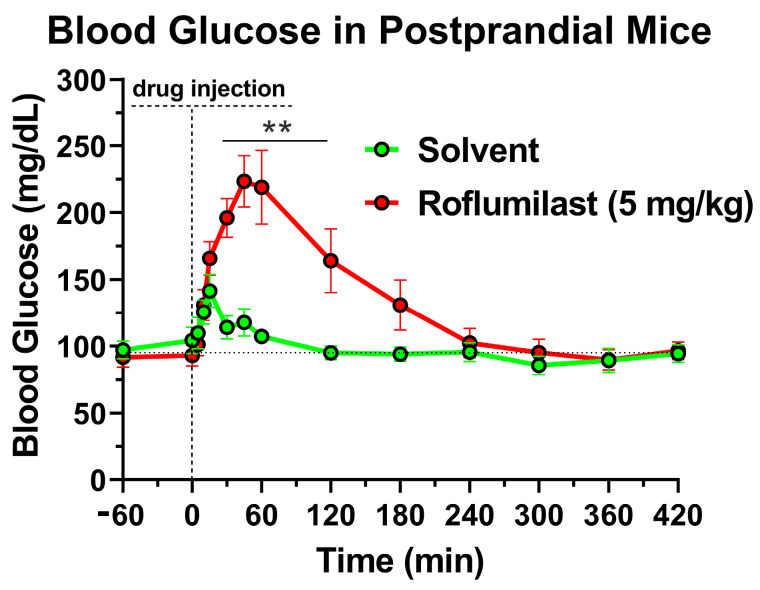
Acute treatment with the PAN-PDE4 inhibitor roflumilast induces a transient increase in postprandial blood glucose levels. The graph shows blood glucose levels, measured with glucometer test strips at tail pricks in postprandial mice that had been deprived of food for 5 h prior to treatment with the PAN-PDE4 inhibitor roflumilast (5 mg/kg; intraperitoneal (i.p.) injection) or solvent controls. The data represent the mean ± SEM of *n* = 8 mice. Roflumilast significantly increased blood glucose levels (**, *p* < 0.01) compared to solvent controls, as determined by a two-way ANOVA with Sidak’s post hoc test.

While exploring the glucose-lowering effects of chronic PDE4 inhibition, as reported previously [28,29], we observed that acute administration of the PAN-PDE4 inhibitor roflumilast (5 mg/kg; via intraperitoneal (i.p.) injection) produced an unexpected increase in blood glucose levels in postprandial C57BL/6 mice, as measured with glucometer test strips on blood drops obtained via tail-vein pricks (Figure 1). The transient rise in blood glucose levels upon the i.p. injection of roflumilast is rapid in onset and is apparent within 15 min. It reaches its maximum at ~45 min after drug administration and gradually returns to baseline levels within 3 to 4 h thereafter (Figure 1).

### 2.2. An Acute Increase in Blood Glucose Levels Is a Class Effect of PAN-PDE4 Inhibitors

**Figure 2:**
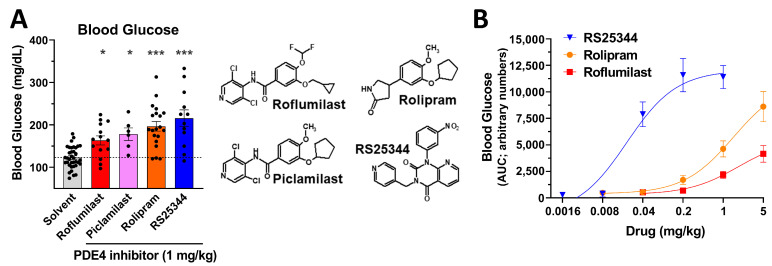
An acute increase in blood glucose levels is a class effect of PAN-PDE4 inhibitors. (**A**) Blood glucose levels in postprandial mice are shown at 30 min after the injection (i.p.) of the PAN-PDE4 inhibitors roflumilast, piclamilast/RP73401, rolipram, and RS25344 (all 1 mg/kg), or solvent controls. The chemical structures of the PDE4 inhibitors are shown for comparison. Data represent the mean ± SEM. Statistical significance was determined using Kruskal–Wallis and Dunn’s post hoc tests and is indicated as * (*p* < 0.05) and *** (*p* < 0.001). (**B**) After measuring baseline blood glucose levels (0 min time point) in postprandial mice, the animals were injected with the indicated doses of RS25344, rolipram, or roflumilast (all i.p.; *n* ≥ 6) and blood glucose levels were measured again at 30, 60, 90, and 120 min after drug injection. Data represent the mean ± SEM and are expressed as the area under the curve (AUC). Dose-response curves for RS25344, rolipram, and roflumilast are significantly different (*p* < 0.01) from each other, as determined by a two-way ANOVA with Sidak’s post hoc test.

To establish whether the transient increase in blood glucose levels induced by roflumilast is truly due to PDE4 inhibition, rather than an off-target effect of roflumilast, we next assessed the acute effects of multiple, structurally distinct PAN-PDE4 inhibitors on blood glucose levels in postprandial mice (Figure 2). The PAN-PDE4 inhibitors rolipram, RS25344, and piclamilast/RP73401, all replicated the effect of roflumilast and elevated blood glucose levels (Figure 2A), thus confirming that transient hyperglycemia is a shared class effect of PDE4 inhibitors.

There is a trend in the order of potencies by which these PDE4 inhibitors elevate blood glucose levels (RS25344 > rolipram > roflumilast; Figure 2A,B), that aligns well with the order of potencies of these drugs to induce several PDE4-dependent physiologic paradigms in mice, such as body temperature regulation or salivation [30,31,32,33], further supporting the idea that the drugs’ effects are mediated via PDE4 inhibition.

### 2.3. PDE4 Controls Blood Glucose Levels, Independent of Its Effects on Body Temperature

**Figure 3:**
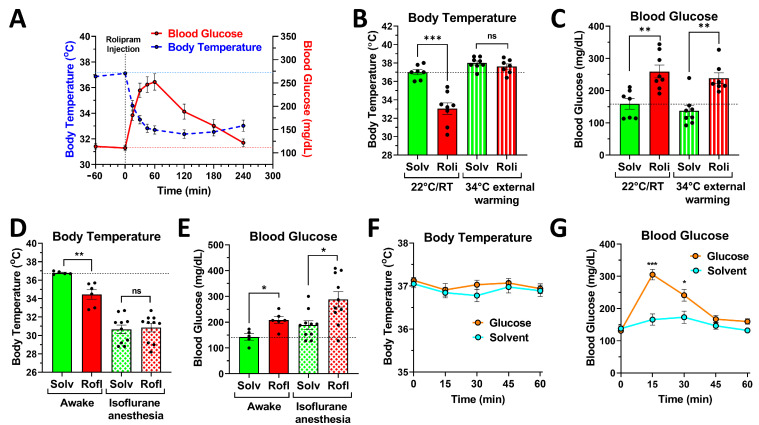
Transient hypothermia and hyperglycemia are independent effects of PAN-PDE4 inhibitor treatment. (**A**) Treatment with a PAN-PDE4 inhibitor induces acute hypothermia, as well as hyperglycemia, on similar time courses. Postprandial mice were injected with the PDE4 inhibitor rolipram (5 mg/kg; i.p.; *n* = 12) and blood glucose levels (red/solid line) and body temperature (blue/striated line) were measured at the indicated time points. (**B**,**C**) External warming prevents PDE4 inhibitor-induced hypothermia, but not increased blood glucose levels. After 5 h of food deprivation, mice were acclimated to cages maintained at room temperature (22 °C; solid bars) or to cages externally warmed to 34 °C using an electric heater (striated bars). Mice were then injected with rolipram (Roli; 5 mg/kg; i.p.) or the solvent control (Solv), and body temperature (**B**) and blood glucose levels (**C**) were assessed 30 min later. (**D**,**E**) Isoflurane anesthesia equalizes body temperatures and locomotion but does not prevent increased blood glucose levels in response to PDE4 inhibitor treatment. Shown are the body temperatures (**D**) and blood glucose levels (**E**) in mice at 30 min after treatment with the PAN-PDE4 inhibitor roflumilast (Rofl; 5 mg/kg; i.p.) or solvent controls (Solv). The effects of drug treatment were compared in awake mice (solid bars) to mice maintained under continuous isoflurane anesthesia beginning at 10 min prior to drug/solvent injection and continued throughout (patterned bars). (**F**,**G**) Elevated blood glucose levels do not cause hypothermia in mice. After an assessment of baseline body temperature and blood glucose levels, postprandial mice were given glucose (2 g/kg in water; oral gavage (o.g.); *n* = 10) or the same volume of water (solvent), and body temperature (**F**) and blood glucose levels (**G**) were measured at the indicated time points. All data represent the mean ± SEM. Statistical significance was determined using Mann–Whitney tests (bar graphs) or a two-way ANOVA with Sidak’s post hoc test (time courses) and is indicated as: ns (*p* > 0.05); * (*p* < 0.05); ** (*p* < 0.01); and *** (*p* < 0.001).

The treatment of mice with PDE4 inhibitors produces several acute physiologic effects, including hypothermia [33,34] and hypokinesia [30,33], which occur with a rapid onset that is similar to the glycemic effects of PDE4 inhibition (see Figure 3A). This raises the possibility that the increase in blood glucose may simply be a consequence of changes in body temperature or muscle contraction, given that both thermogenesis (which is likely to be suspended in hypothermic mice) and muscle contraction (which is reduced in hypokinetic mice) are energy-intensive processes. We employed two independent approaches to exclude this possibility, and to demonstrate that the glycemic effects of PDE4 inhibitors are independent of the drugs’ effects on body temperature and/or muscle contraction (locomotor activity).

In a first approach, mice were maintained in cages externally warmed to 34 °C, which stabilized the body temperature of the mice and precluded the PDE4 inhibitor-induced hypothermia observed in mice kept at room temperature (Figure 3B). While warming the mice completely prevented drug-induced hypothermia (Figure 3B), treatment with PDE4 inhibitors remained effective in increasing blood glucose levels in mice maintained at 34 °C (Figure 3C), suggesting that the regulation of body temperature and blood glucose levels are independent effects of PAN-PDE4 inhibition.

In a second approach, we utilized isoflurane anesthesia to abrogate the differences in body temperature and muscle contraction between the hypokinetic and hypothermic PDE4 inhibitor-treated mice and the solvent control group. Anesthesia is known to critically inactivate cold-defense mechanisms in mice and humans alike, thus resulting in fast-onset hypothermia [33,35]. As shown previously [33], PDE4 inhibition appears to lower the cold-defense set point of mice, rather than completely suspending body temperature regulation. In other words, the body temperature of PDE4 inhibitor-treated mice will decrease until it reaches the new cold-defense set-point, at which point the heat-generating/preserving mechanisms are re-activated to maintain body temperature at this new, lower level (e.g., at 34 °C, instead of the normal 37 °C). Because the induction of isoflurane anesthesia reduces body temperatures to significantly lower levels than PDE4 inhibitor treatment (~30 °C after isoflurane anesthesia, compared to 34 °C after roflumilast treatment (Figure 3D)), PDE4 inhibition has no further effects on body temperature in anesthetized mice (Figure 3D). Moreover, anesthesia also ablates any differences in skeletal muscle contraction/activity between the PDE4 inhibitor- and solvent control groups. As seen with external warming, PDE4 inhibition produced a significant increase in blood glucose levels in mice maintained under isoflurane anesthesia (Figure 3E), suggesting that the glycemic effects of PDE4 inhibitors are independent effects and do not result from altered muscle contraction and/or body temperature regulation. Additionally, increased blood glucose levels are also unlikely to be the cause of PDE4 inhibitor-induced hypothermia, given that increasing blood glucose levels per se (such as via oral gavage of a glucose solution) does not induce hypothermia in mice (Figure 3F,G).

### 2.4. The Effect of PDE4 Inhibition on Blood Glucose Levels Is Independent of Changes in Insulin Secretion or Insulin Sensitivity

**Figure 4:**
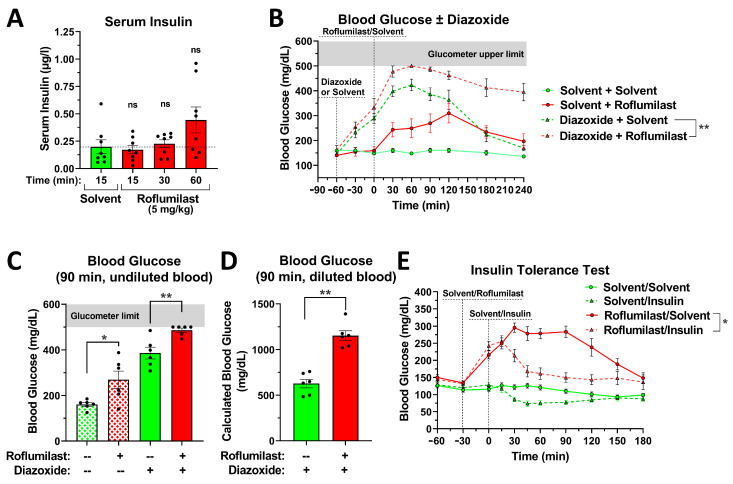
The acute glycemic effects of PDE4 inhibition are not driven by changes in insulin secretion or tissue insulin sensitivity. (**A**) PDE4 inhibition does not lower serum insulin levels. Shown are insulin levels measured in serum of postprandial mice that had been cheek-bled at the indicated times after injection of the PDE4 inhibitor roflumilast (5 mg/kg; i.p.) or solvent control. (**B**–**D**) Inhibition of insulin release with diazoxide elevates blood glucose levels via a mechanism that is distinct and additive to that of PDE4 inhibition. After 5 h of food deprivation, mice were injected with diazoxide (25 mg/kg; i.p.; *n* = 6; −60 min time point) or solvent control, followed 60 min later by a second injection with either the PAN-PDE4 inhibitor roflumilast (5 mg/kg; i.p.; 0 min time point) or solvent control. The glucometer used to measure blood glucose at tail pricks does not read above the upper limit of 500 mg/dL glucose that is indicated by the grey-shaded area. Treatment with roflumilast significantly (*p* < 0.01) increased blood glucose levels compared to solvent controls, in both the absence and presence of diazoxide. In (**C**,**D**), blood glucose levels measured at 90 min after PDE4 inhibitor treatment in the same mice are reported. (**C**) shows the blood glucose levels measured in undiluted blood directly at the mouse tail-vein prick, leading to 4 out of 6 mice in the “diazoxide/roflumilast” group reading at the detection limit of 500 mg/dL. In (**D**), shown are calculated blood glucose levels for the diazoxide-treated groups that were measured after diluting 1 µL of tail-vein blood 1:5 with PBS, thus obtaining readings in the normal range of the glucometer. (**E**) PDE4 inhibition does not impair insulin sensitivity. After 5 h of food deprivation, mice were injected with the PDE4 inhibitor roflumilast (5 mg/kg; i.p.; *n* ≥ 10; −30 min time point) or solvent control, followed 30 min later with an injection of insulin (Humulin R; i.p.; 0 min time point) or solvent control. Shown are blood glucose measurements at tail-vein pricks at the indicated time points. The injection of insulin induced a significant reduction (*p* < 0.001) in blood glucose levels, in both the absence and the presence of roflumilast. All data represent the mean ± SEM. Statistical significance was determined using the Mann–Whitney test (bar graphs comparing two groups) and the Kruskal–Wallis test, followed by Dunn’s post hoc test to determine the differences between more than two treatment groups or by a two-way ANOVA with Sidak’s post hoc test (time courses), and is indicated as ns (*p* > 0.05); * (*p* < 0.05); and ** (*p* < 0.01).

**Figure 5:**
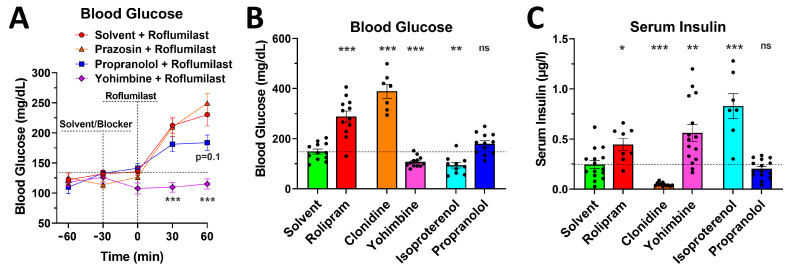
Pretreatment with the β-blocker propranolol or the α_2_-adrenoceptor blocker yohimbine alleviates the glycemic effects of PAN-PDE4 inhibition. (**A**) Postprandial mice were injected (i.p.) with the β-blocker propranolol (5 mg/kg), the α_1_-adrenoceptor blocker prazosin (1 mg/kg), the α_2_-adrenoceptor blocker yohimbine (5 mg/kg), or solvent controls, followed 30 min later with an injection of the PDE4 inhibitor roflumilast (5 mg/kg), in all mice. Blood glucose levels measured at tail pricks are shown at the indicated time points. (**B**,**C**) Postprandial mice were injected with the PDE4 inhibitor rolipram (5 mg/kg), the α_2_-adrenoceptor agonist clonidine (1 mg/kg), the α_2_-adrenoceptor blocker yohimbine (5 mg/kg), the β-agonist isoproterenol (5 mg/kg), the β-blocker propranolol (5 mg/kg), or solvent controls, followed 60 min later by the measurement of blood glucose levels at tail pricks (**B**) and subsequent cheek bleeds to assess serum insulin levels via an ELISA (**C**). All data represent the mean ± SEM. Statistical significance was determined using the Mann–Whitney test (bar graphs) or a two-way ANOVA with Sidak’s post hoc test (time courses), and is indicated as ns (*p* > 0.05); * (*p* < 0.05); ** (*p* < 0.01); and *** (*p* < 0.001).

Insulin is the principal hormone responsible for preventing hyperglycemia, raising the question of whether PDE4 inhibition may elevate blood glucose levels by lowering serum insulin levels (e.g., reducing insulin release) or reducing the sensitivity of metabolic/peripheral tissues to insulin. As shown in Figure 4A, treatment with the PDE4 inhibitor roflumilast does not reduce serum insulin levels when measured at 15 min or 30 min after drug application, the same period during which roflumilast induces a rapid rise in serum glucose levels (Figure 1). This suggests that this transient hyperglycemia is not primarily driven by reduced insulin release. Serum insulin levels tend to increase at 60 min after roflumilast injection (Figure 4A; see also Figure 5C for a similar effect with rolipram), likely as a feedback response to elevated blood glucose levels.

To further confirm that PDE4 inhibition does not act by inhibiting insulin release, we explored the co-treatment of a PDE4 inhibitor with diazoxide. Diazoxide is a drug used to treat hypoglycemia, which acts by opening ATP-sensitive potassium channels on pancreatic β-cells to potently inhibit insulin release, thus increasing blood glucose levels [36]. As shown in Figure 4B–D, while diazoxide or PDE4 inhibitor treatment each increases blood glucose levels on its own, their combination produces additive effects, suggesting that each drug acts via a different mechanism. The dramatic increase in blood glucose levels produced by the combination of diazoxide and roflumilast is underestimated in Figure 4B because the glucometer strips used to assess blood glucose levels are limited to a maximum blood glucose reading of 500 mg/dL. At the 60-min time point, the blood samples of the 6 mice in the “diazoxide + roflumilast” group were all measured at the upper limit of 500 mg/dL of glucose. At the subsequent 90-min time point, we then measured blood glucose twice: (1) we measured glucose levels in a blood drop directly at the tail of the mouse (Figure 4C), and (2) we diluted a microliter of tail blood 1:5 in PBS prior to analysis, thereby bringing the blood glucose readings within the normal range of the glucometer (Figure 4D). As shown in Figure 4D, after correcting for the 1:5 dilution, the measured glucose levels for the “diazoxide only” group and the “diazoxide + roflumilast” group clearly differed, suggesting that PDE4 inhibitors increase blood glucose levels via a mechanism that was distinct from the inhibition of insulin release induced by diazoxide.

Finally, to assess whether PDE4 inhibitor treatment may affect the tissue sensitivity to insulin, insulin tolerance tests were performed. To this end, mice were first injected with either roflumilast or solvent control, followed by the administration of insulin 30 min later. As shown in Figure 4E, in solvent control mice, insulin injection produces a significant reduction in blood glucose levels (green striated line) compared to control mice not injected with insulin (green solid line). At the time of insulin injection (0 min time point), blood glucose levels are much higher in roflumilast-treated mice (red lines), but insulin injection still potently reduced blood glucose levels (red striated line) compared to mice not treated with insulin (red solid line). Together, these data suggest that treatment with PDE4 inhibitors does not reduce insulin secretion, nor does it reduce whole-body insulin sensitivity to elevated blood glucose levels.

### 2.5. Blockade of α_2_- or β-Adrenoceptors Alleviates the Acute Glycemic Effects of PAN-PDE4 Inhibition

To begin elucidating the molecular pathways whereby PDE4 inhibition increases blood glucose levels, we explored its dependence on adrenoceptor signaling. To this end, the effect of PDE4 inhibition on blood glucose levels was assessed in mice pretreated with various adrenoceptor blockers (Figure 5A). In addition, we contrasted the effects of a 60-min treatment with the agonists and antagonists of α_2_- and β-adrenoceptors on both blood glucose levels (Figure 5B) and serum insulin levels (Figure 5C) with the effects of the PDE4 inhibitor roflumilast.

As shown in Figure 5A, pretreatment with the α_1_-adrenoceptor antagonist prazosin did not substantially alter PDE4 inhibitor-induced hyperglycemia (*p* > 0.05). Conversely, pretreatment with the α_2_-adrenoceptor antagonist yohimbine completely abrogated the effect of PAN-PDE4 inhibition. While α_2_-adrenoceptors are expressed at multiple sites in both the central nervous system and peripheral organs, the principal site of α_2_-adrenoceptor action in the regulation of blood glucose levels is thought to lie in the control of insulin secretion by pancreatic β-cells [37,38,39,40]. As shown in Figure 5B,C, treatment with the α_2_-agonist clonidine indeed potently lowered serum insulin levels (Figure 5C) and caused elevated blood glucose levels as a result (Figure 5B). Conversely, the α_2_-adrenceptor blockade with yohimbine increased serum insulin levels and suppressed baseline blood glucose. Treatment with yohimbine, and the resulting elevation of serum insulin levels, completely prevented PDE4 inhibitor-induced hyperglycemia (Figure 5A), thus further demonstrating that PDE4 inhibition does not reduce whole-body insulin sensitivity but acts via an insulin-independent mechanism to elevate blood glucose levels.

PDE4s have been shown to closely regulate β-adrenergic signaling across a variety of cell types (from cardiac myocytes to fibroblasts or epithelial cells) and physiologic paradigms (from cardiac contraction to salivation) [9,31,41,42,43,44]. In most scenarios, PDE4 enzymes limit β-adrenoceptor-induced cAMP signals; consequently, PDE4 inhibition results in an initial magnification of β-adrenoceptor signaling. It is worth noting that, in postprandial mice, the activation of β-adrenoceptor signaling with isoproterenol produces opposite effects on blood glucose, compared to PDE4 inhibition, with isoproterenol treatment reducing, rather than increasing, blood glucose levels (Figure 5B). This difference is probably due to the fact that β-adrenoceptor agonists act on pancreatic β-cells to promote insulin release (Figure 5C), whereas PDE4 inhibitors do not. Despite this difference in the glycemic effects of isoproterenol and PDE4 inhibitors, pre-treatment with the β-blocker propranolol alleviates the glycemic effects of PDE4 inhibitors, suggesting that PDE4 inhibition may act, at least in part, by amplifying β-adrenoceptor-dependent cAMP signals in metabolic tissues other than the pancreatic β-cell.

### 2.6. PDE4 Inhibition Induces Glycemic Effects in Mice Deprived of Food for 5 h or for 16 h Overnight

**Figure 6:**
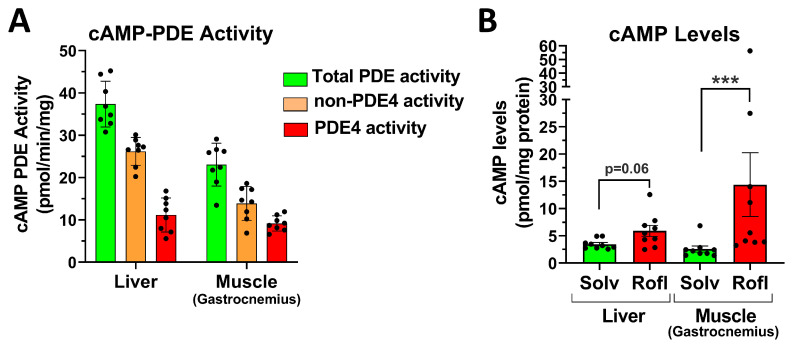
PDE4 activity and cAMP levels in metabolic tissues. (**A**) Detergent extracts, prepared from mouse liver and skeletal muscle (gastrocnemius), were subjected to in vitro cAMP-PDE activity assays in the presence or absence of the PDE4 inhibitor rolipram (10 µM). Total cAMP-PDE activity is defined as the rate of cAMP hydrolysis measured in the absence of rolipram, whereas PDE4 and non-PDE4 activity are defined as the fraction of total activity that is either inhibited or that is insensitive to inhibition by rolipram, respectively. (**B**) Shown are cAMP levels in the tissues of postprandial mice at 30 min after treatment with the PAN-PDE4 inhibitor roflumilast (5 mg/kg; i.p.) or solvent control (Solv). Data represent the mean ± SEM. Statistical significance was determined using the Mann-Whitney test and is indicated as *** (*p* < 0.001).

The four genes that comprise the PDE4 family (PDE4A to D) are widely expressed throughout the body and are found in many cells and tissues, including those preeminently involved in the control of blood glucose levels. This includes skeletal muscles and the liver, in which PDE4 isoenzymes comprise about 40% and 30% of the total cAMP-hydrolytic capacity, respectively (Figure 6A). Consequently, acute treatment with PAN-PDE4 inhibitors increases cAMP signaling in a large number of cells and tissues, including skeletal muscle and the liver (Figure 6B), which may then directly or indirectly affect blood glucose levels.

**Figure 7:**
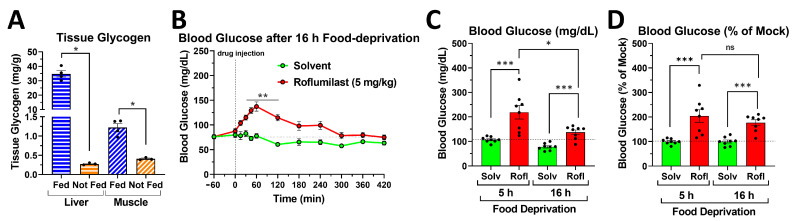
Glucose release from hepatic glycogen is not required for the glycemic effects of PAN-PDE4 inhibitors. (**A**) The glycogen levels in the liver and skeletal muscle (gastrocnemius) of fed mice (which were given 4 g/kg glucose o.g. at 60 min prior to euthanasia and tissue extraction) and of mice deprived of food for 16 h overnight (Not Fed) are shown. (**B**–**D**) Mice were food-deprived for 16 h overnight prior to treatment with either the PAN-PDE4 inhibitor roflumilast (Rofl; 5 mg/kg; i.p.; *n* = 8) or solvent control. (**B**) Shown is the complete time course of blood glucose levels in mice deprived of food for 16 h overnight after treatment with roflumilast or solvent control. (**C**,**D**) Comparison of blood glucose levels at 60 min after treatment with roflumilast or solvent control (Solv) in mice food-deprived for 5 h (data extracted from Figure 1) or in mice food-deprived for 16 h overnight (data extracted from Figure 7B). Data are reported as either the amount of blood glucose in mg/dL(**C**) or as a percentage of the solvent control (**D**). All data represent the mean ± SEM. Statistical significance was determined using the Mann–Whitney test (bar graphs) or a two-way ANOVA with Sidak’s post hoc test (time courses) and is indicated as ns (*p* > 0.05); * (*p* < 0.05); ** (*p* < 0.01); and *** (*p* < 0.001).

In the absence of food intake, blood glucose levels represent the equilibrium between the rate of glucose production by the liver, kidney, and intestines and the combined rate of glucose uptake and utilization by all organs of the body. In the postprandial phase, hepatic glycogenolysis is a major source of the glucose released into the bloodstream to maintain blood glucose levels. Conversely, after an extended fasting period, the hepatic glycogen pool becomes depleted, and gluconeogenesis replaces glycogenolysis as the major source of glucose production. Indeed, as shown in Figure 7A, compared to the hepatic glycogen content of fed mice, only ~1% of hepatic glycogen remains in mice deprived of food for 16 h overnight. Hepatic glycogenolysis during the postprandial phase is driven predominantly by glucagon, which works via the activation of a G_s_-coupled receptor and increased cAMP/PKA (protein kinase A) signaling to activate glycogen phosphorylase and inhibit glycogen synthase. To test whether increased cAMP-dependent glycogenolysis is a predominant driver of the glycemic effects of PDE4 inhibitors, we tested the effects of PDE4 inhibitors in mice deprived of food for 16 h overnight, in which hepatic glycogen stores are depleted. As shown in Figure 7B, treatment with roflumilast still potently increased blood glucose levels in mice deprived of food overnight, suggesting that the glycemic effects of PDE4 inhibitors do not strictly depend on increased liver glycogenolysis. The maximal blood glucose levels induced by PDE4 inhibitors are higher in postprandial mice deprived of food for 5 h (~220 mg/dL; Figure 1) than in mice deprived of food for 16 h (~140 mg/dL Figure 7B), but so are the baseline blood glucose levels (0 min time point, or solvent controls; Figure 7C). Plotting the PDE4 inhibitor-induced blood glucose levels as a percentage over solvent controls (Figure 7D) indicates that the glycemic effects of PDE4 inhibitors in postprandial mice and in mice that were deprived of food overnight are comparable, suggesting that they are not strictly dependent upon increased hepatic glycogenolysis.

### 2.7. PDE4 Inhibition Promotes Skeletal Muscle Glycogenolysis in the Face of Reduced Glucose Uptake

**Figure 8:**
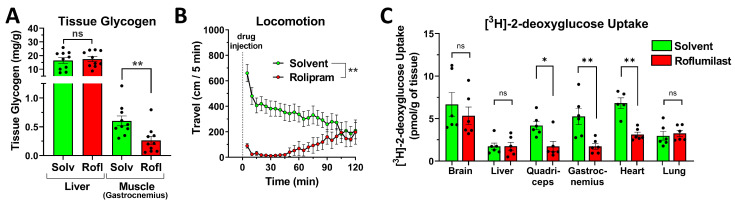
PDE4 inhibition induces glycogenolysis in skeletal muscle and inhibits glucose uptake in skeletal muscle and heart. (**A**) PDE4 inhibition induces glycogenolysis in skeletal muscle. The glycogen levels in the liver and skeletal muscle (gastrocnemius) in postprandial mice are shown after treatment with the PAN-PDE4 inhibitor roflumilast (Rofl; 5 mg/kg; i.p.) or solvent control (Solv) for 90 min. Data are expressed as mg of glycogen per g of tissue weight. (**B**) PAN-PDE4 inhibition reduces locomotion, as reflected by reduced travel distance. Mice were injected with the PDE4 inhibitor rolipram (3 mg/kg; i.p.; *n* = 6) or solvent control, placed immediately in a new cage, and then locomotion was assessed using SmartCageTM technology. Traces represent changes in travel distance (cm per 5 min interval). (**C**) Postprandial mice were injected with the PDE4 inhibitor roflumilast (5 mg/kg; i.p.) or solvent control, followed 15 min later by injection with [^3^H]-2-deoxyglucose (intravenous, i.v.). Animals were euthanized 30 min after the tracer injection, their tissues were homogenized, and the phosphorylated [^3^H]-2-deoxyglucose-6-phosphate was isolated by anion exchange chromatography and quantified by scintillation counting. Data are expressed as pmol [^3^H]-2-deoxyglucose-6-phosphate per g of tissue wet weight. All data represent the mean ± SEM. Statistical significance was determined using the Mann–Whitney test (bar graphs) or a two-way ANOVA with Sidak’s post hoc test (time courses) and is indicated as ns (*p* > 0.05), * (*p* < 0.05), and ** (*p* < 0.01).

The idea that hepatic glycogenolysis is not a primary driver of the glycemic effects of PDE4 inhibition is further supported by the observation that a 90-min treatment with a PDE4 inhibitor—while substantially increasing blood glucose levels—did not lead to a significant reduction in hepatic glycogen content, compared to solvent controls (Figure 8A). Intriguingly, the glycogen content of skeletal muscle was, however, reduced over the same time period. This reduction in muscle glycogen is even more surprising, given that PDE4 inhibition produces profound hypokinesia in mice (Figure 8B), which would suggest a reduced demand for energy in support of muscle contractions in PDE4 inhibitor-treated mice, compared to the solvent controls. To further delineate the role of skeletal muscle in glucose homeostasis, we next performed 2-deoxyglucose uptake studies. To this end, postprandial mice that had been treated with the PDE4 inhibitor roflumilast or solvent control were injected with [^3^H]-2-deoxyglucose, and the uptake of the tracer into tissues was quantified. As shown in Figure 8C, treatment with roflumilast significantly reduced 2-deoxyglucose uptake in muscle tissues, including the gastrocnemius, quadriceps, and heart, while the uptake into the brain, liver, and lung, which were included as non-muscle-control tissues, was unchanged. Together, these data indicate that the reduced uptake of blood glucose into skeletal muscle is a major driver of the glycemic effects of acute PDE4 inhibitor treatment.

## 3. Discussion

### 3.1. An Acute, Transient Increase in Blood Glucose Levels Is a Class Effect of PAN-PDE4 Inhibitors in Mice

In this study, we report that the acute, PAN-selective inhibition of PDE4s induces a transient increase in blood glucose levels in mice (Figure 1, Figure 2 and Figure 3). Blood glucose levels rapidly rise upon the i.p. injection of a PDE4 inhibitor (within 15 min), peak at ~45–60 min after injection, and return to basal levels within 3 to 4 h after drug treatment (Figure 1 and Figure 3A). These glycemic effects are dose-dependently induced by structurally distinct drugs, including the archetypal PDE4 inhibitor rolipram and the clinically used inhibitor roflumilast, as well as piclamilast/RP73401 and RS25344 (Figure 2), suggesting that this is a class effect of PAN-selective PDE4 inhibitors.

The PDE4 family is the largest of the 11 families of mammalian PDEs, comprising four genes, PDE4A-D, which, in turn, encode for over 25 protein variants. These are widely expressed throughout the body, with at least one of the four PDE4 genes being expressed in, essentially, every cell. As a result, there is a large number of possible locations—tissues, cells, and subcellular compartments—wherein PDE4s may serve to control blood glucose levels, and which will be of interest to delineate in future studies. Our initial characterization, presented here, suggests the following findings.

### 3.2. PDE4 Inhibition Does Not Reduce Insulin Secretion and/or Sensitivity

Insulin is the master regulator of glucose homeostasis and is tasked with protecting organisms from elevated blood glucose levels. Indeed, hyperglycemia has become somewhat synonymous with reduced insulin secretion or sensitivity, given the prevalence of type I and type II diabetes, respectively. Treatment with PDE4 inhibitors also induces hyperglycemia; however, several lines of evidence suggest that the glycemic effects of PDE4 inhibitors are not predominantly driven by changes in either insulin secretion or signaling.

First, it is well-established that insulin secretion from pancreatic β-cells is positively regulated by cAMP signaling, and, thus, the inhibition of any PDE would be expected to increase insulin secretion and, thereby, lower blood glucose levels—an outcome in opposition to the transient hyperglycemic effects of PDE4 inhibitors that we report here. The cAMP-dependent regulation of insulin secretion is illustrated by the observation that clonidine, which activates G_i_-coupled α_2_-adrenoceptors and thus lowers intracellular cAMP, reduces serum insulin levels, and leads to an increase in blood glucose (Figure 5B,C) [37]. Conversely, the blockade of α_2_-adrenoceptors with yohimbine, or the stimulation of G_s_-coupled β-adrenoceptors with isoproterenol, increases serum insulin levels and subsequently lowers blood glucose (see Figure 5B,C).

Second, PDE4 has been shown to be expressed in the rat insulinoma cell line INS-1 and primary human β-cells. However, PDE1 and PDE3, not PDE4, appear to be the primary regulators of insulin secretion and, as mentioned earlier, increased cAMP signaling resulting from PDE inhibition within β-cells should be promoting insulin release, rather than inhibiting it [7,45,46,47].

Third, blood glucose levels rapidly rise during the first 30 min after PDE4 inhibitor injection (Figure 1 and Figure 3A), while serum insulin levels remain unchanged (Figure 4A), suggesting that PDE4 inhibition also does not indirectly affect insulin secretion (e.g., via neuronal- or incretin-dependent effects).

Fourth, treatment with diazoxide potently induces hyperglycemia in mice, but its effects are additive to the glycemic effects of PDE4 inhibition (Figure 4B–D), suggesting that the two drugs act via distinct mechanisms. That is, diazoxide acts by opening ATP-sensitive potassium channels on pancreatic β-cells, thereby inhibiting insulin secretion [36], suggesting that PDE4 inhibitors must increase blood glucose levels by a mechanism distinct from inhibiting insulin secretion.

Fifth, in the presence of normal serum insulin levels, hyperglycemia may result from the reduced sensitivity of peripheral tissues to insulin signaling, such as the reduced uptake of glucose into the liver, skeletal muscle, or fat tissue. However, as demonstrated in the insulin tolerance tests shown in Figure 4E, the injection of insulin is highly effective in reducing blood glucose levels in mice treated with PAN-PDE4 inhibitors, suggesting that the glycemic effects of PAN-PDE4 inhibition are not mediated via reduced whole-body insulin sensitivity.

Finally, the increase in blood glucose levels upon PDE4 inhibitor treatment peaks at about 45 to 60 min after drug injection (Figure 1 and Figure 3A). At this time point, we begin to observe elevated levels of serum insulin in response to treatment with either roflumilast (Figure 4A) or rolipram (Figure 5C), suggesting that the PDE4 inhibitor-induced increase in blood glucose levels triggers the appropriate glucose-induced insulin secretion by pancreatic β-cells. We consider this increase in insulin secretion an important factor to explain why blood glucose levels plateau at ~60 min after PDE4 inhibitor injection and then promptly return to baseline levels, thereby quenching the glycemic effects of PDE4 inhibition. As shown in Figure 3A, there is a clear difference between the hypothermia and hyperglycemia measured upon PDE4 inhibitor treatment in the same mice, in that blood glucose levels return to baseline levels, while body temperatures remain low for an extended time period. The latter suggests that drug clearance does not occur rapidly; instead, compensatory mechanisms, such as increased insulin secretion (Figure 4A and Figure 5C), rapidly counter the glycemic effects of PDE4 inhibitors and promote the return of blood glucose levels to their nadir.

### 3.3. PDE4 Inhibition and Glucose Production and Utilization

While all mammalian cells may utilize glucose, only a limited number of tissues, primarily the liver, and to minor extents the kidneys and intestines [48], are equipped to release glucose back into the bloodstream upon its release from intracellular stores (glycogenolysis) or via de novo synthesis (glucogenesis). Increased cAMP signaling in these tissues, such as upon glucagon stimulation, is well known to promote glucose production via the posttranslational regulation of key enzymes in glycogenolysis (glycogen phosphorylase and synthase [8]) and gluconeogenesis (phosphofructokinase 2, pyruvate kinase [49]). This, therefore, provides a rationale whereby the inhibition of cAMP hydrolysis by a PDE may increase blood glucose levels via increased glucose production and release. In postprandial mice, treatment with PDE4 inhibitors did not reduce the hepatic glycogen content (Figure 8A). In addition, PDE4 inhibition potently increased blood glucose levels also in mice deprived of food for 16 h overnight, in which the hepatic glycogen levels were depleted (Figure 7B–D). These findings suggest that the glycemic effects of PDE4 inhibitors do not strictly depend on increased hepatic glycogenolysis. However, future studies will have to address whether increased glucose production via gluconeogenesis (rather than glycogenolysis) may contribute to the glycemic effects of PDE4 inhibitors.

Conversely, the current data demonstrate that reduced glucose uptake and utilization, in particular by skeletal muscle, is a significant contributor to the glycemic effects of PDE4 inhibitors. The most direct evidence is provided by the observation that treatment with PDE4 inhibitors reduces the uptake of 2-deoxyglucose specifically into muscle tissues, whereas its uptake into the brain, liver, and lung is unchanged (Figure 8C). Intriguingly, the effect of PDE4 inhibition on glucose uptake has been studied in distinct models before, with somewhat varying results. These prior studies have shown that treatment with the PDE4 inhibitor rolipram reduced insulin-stimulated glucose uptake in isolated perfused rat heart [50], as well as the [^3^H]-deoxyglucose uptake in mouse brain and heart in vivo [51]. Conversely, in some ex vivo models, treatment with rolipram increased, rather than decreased, the glucose uptake including in mouse soleus muscle [52] or rat L6 myotubes [53]. This suggests that experimental conditions may have a critical impact on the effect of PDE4 inhibition on glucose uptake, including the environment provided in ex vivo experiments, but perhaps also differences in animal species/strain, nutrition state, etc. As an intriguing illustration of the significance of experimental conditions, prior studies have shown that the treatment of rats with the β-agonist isoproterenol reduces blood glucose levels in postprandial rats (similar to the effect observed here in mice; Figure 5B), but potently increases blood glucose levels in rats fasted overnight [54].

Skeletal muscle is a major site of postprandial glucose disposal and, thus, plays a key role in whole-body glucose homeostasis. It is thus reasonable to propose that the PDE4 inhibitor-induced reduction in muscle glucose uptake is one of the drivers of the elevated blood glucose levels in the animals, while not excluding the potential roles of other cells/tissues (e.g., the heart (Figure 8C) and fat deposits). How PDE4 inhibition reduces muscle glucose uptake remains to be established. By controlling the membrane localization of glucose transporter type 4 (GLUT4), the main glucose transporter in skeletal muscle, insulin acts as the primary regulator of skeletal muscle glucose uptake. However, as previously discussed, PDE4 inhibition does not appear to interfere with the anti-glycemic effects of insulin (Figure 4E). Several prior studies proposed a direct, cAMP-mediated inhibition of GLUT4-mediated glucose uptake [55,56,57] that is independent of the mobilization of GLUT4 to the plasma membrane; this represents a potential mechanism to be explored. Considering that muscle glycogen is rapidly depleted in PDE4 inhibitor-treated mice, despite the apparent hypokinesia, and, hence, a lack of energy demand from muscle contractions, we propose an additional, unifying mechanism for our observations. cAMP signaling is well established to promote glycogenolysis, while inhibiting glycogen synthesis, which we observe in skeletal muscle (Figure 8A) via the PKA-mediated activation of phosphorylase kinase and protein phosphatase 1 inhibitor, which, in turn, synergize to activate glycogen phosphorylase and inactivate glycogen synthase [2,8]. Given that PDE4 inhibitor-treated mice do not move their muscles and may, thus, have limited demand for energy production, they may not utilize the glucose-6-phosphate that is produced by the hexokinase-mediated phosphorylation of glucose entering the cell [58,59,60,61] or by the phosphoglucomutase-mediated interconversion of glucose-1-phosphate that results from glycogen breakdown [62], leading to glucose-6-phosphate accumulation in the myocyte. Glucose-6-phosphate is a potent allosteric inhibitor of hexokinases Types I and II, the two main hexokinase enzymes expressed in skeletal muscle [59,60,61,63,64]. With the hexokinases inhibited by glucose-6-phosphate, glucose entering the muscle cell may no longer be phosphorylated and thus trapped in the cell and may instead leave the cells via GLUT-assisted transport, thus reducing the overall uptake of glucose into the myocyte. 

Based on the observation that PDE4 contributes a large portion of cAMP-hydrolytic capacity in skeletal muscle (Figure 6A), the significant increase in skeletal muscle cAMP levels upon PDE4 inhibitor treatment (Figure 6B), the reduction in skeletal muscle glycogen levels (Figure 8A), as well as the inhibition of 2-deoxyglucose uptake into muscles upon PDE4 inhibitor treatment in mice (Figure 8C), it is tempting to suggest that inhibition of the PDE4 expressed in myocytes leads to reduced uptake and utilization of glucose by skeletal muscles, and causes the transient increase in blood glucose levels observed upon PDE4 inhibitor treatment. However, at present, we cannot yet exclude the possibility that the PDE4, which is responsible for the glycemic effects of PDE4 inhibitors, may, in small or large parts, reside outside of skeletal muscles and may even exert its effects on muscle function via neuronal, hormonal, and/or metabolic mediators. This possibility remains to be explored using approaches to selectively inactivate PDE4 in skeletal muscle (or other cells/tissues) in future studies.

### 3.4. Clinical Relevance of the Acute Glycemic Effects of PDE4 Inhibitors

While acute PDE4 inhibition in mice induces a transient increase in blood glucose levels, this does not necessarily suggest a pathologic effect or outcome but may reflect a normal physiologic response to elevated cAMP levels (e.g., in skeletal muscle and, potentially, other cells/tissues) for two reasons. First, the effect induced by PDE4 inhibitors in mice does not exceed a normal variation of blood glucose levels, such as the elevation of blood glucose levels upon feeding (see Figure 3G for a comparison). Second, similar to blood glucose levels after feeding, the effect of PDE4 inhibition is transient (see Figure 1 and Figure 3A), and blood glucose levels return to baseline by virtue of glucose-induced insulin secretion (Figure 4A and Figure 5C). Thus, the pattern of glycemic effects induced by PDE4 inhibitors is very different, compared to the chronic elevation of blood glucose levels resulting from impaired insulin secretion and/or signaling as a result of diabetes.

To our knowledge, an acute increase in blood glucose levels upon PDE4 inhibitor treatment has not been reported before in the literature. Thus, it remains to be established whether the acute glycemic effects that we report here in mice are shared by other mammalian species, most importantly, humans. While PAN-PDE4 inhibitors, such as roflumilast, have been used clinically for several years and in thousands of patients, there are no related reports of acute increases in blood glucose levels in humans to date. There are multiple possible explanations for this divergence. Blood glucose levels may not have been measured shortly after drug application in humans. Alternatively, given the significant differences in the basal metabolic rate between mice and humans [65,66], this finding may indicate species differences. Finally, it may simply reflect differences in the drug doses used in our present study in mice, compared to the significantly lower drug doses that are used clinically. PAN-PDE4 inhibitors produce a range of therapeutic benefits, including potent anti-inflammatory effects, as well as cognition- and memory-enhancing benefits [9,15,16,17]. However, this class of drugs is also associated with a number of characteristic adverse effects, principally nausea, diarrhea, and emesis, which narrow the therapeutic window and limit the doses that can be used clinically. For example, roflumilast is prescribed at individual doses of 0.5 mg/tablet in COPD patients. Thus, potentially, the serum levels required to produce a noticeable acute increase in blood glucose levels (see Figure 2) may simply not be reached in patients.

Intriguingly, however, chronic/long-term administration of the PDE4 inhibitor roflumilast has been shown to lower blood glucose levels in multiple independent studies, including in mouse models of obesity and diabetes, as well as in humans [28,29,67]. This effect of PDE4 inhibition is paralleled by, and is perhaps dependent upon, elevated mitochondrial biogenesis and the resulting increase in energy expenditure [28]. It is tempting to speculate whether there may be a causal connection between the acute glycemic effects reported here, eventually driving compensatory mechanisms that promote improved glucose handling in the long term, which remains to be tested experimentally.

## 4. Materials and Methods

### 4.1. Drugs

Roflumilast(3-(cyclopropylmethoxy)-N-(3,5-dichloropyridin-4-yl)-4-(difluoromethoxy)benzamide, MW = 403 g/mol; 1 mg/kg ≈ 2.5 µmol/kg), rolipram (4-(3-cyclopentyloxy-4-methoxyphenyl)pyrrolidin-2-one; MW = 275 g/mol; 1 mg/kg ≈ 3.6 µmol/kg), piclamilast (RP73401; 3-(Cyclopentyloxy)-N-(3,5-dichloropyridin-4-yl)-4-methoxybenzamide; MW = 381 g/mol; 1 mg/kg ≈ 2.6 µmol/kg), prazosin ([4-(4-amino-6,7-dimethoxyquinazolin-2-yl)piperazin-1-yl]-(furan-2-yl)methanone, monohydrochloride; MW = 420 g/mol; 1 mg/kg ≈ 2.4 µmol/kg), clonidine (N-(2,6-dichlorophenyl)-4,5-dihydro-1H-imidazol-2-amine, monohydrochloride; MW = 267 g/mol; 1 mg/kg ≈ 3.7 µmol/kg), yohimbine (17α-hydroxy-yohimban-16α-carboxylic acid, methyl ester, monohydrochloride, MW = 391 g/mol; 1 mg/kg ≈ 2.6 µmol/kg), and diazoxide (7-chloro-3-methyl-1,1-dioxide-2H-1,2,4-benzothiadiazine; MW = 231 g/mol; 1 mg/kg ≈ 4.3 µmol/kg) were sourced from Cayman Chemical (Ann Arbor, MI), isoprenaline/isoproterenol (4-[1-hydroxy-2-(propan-2-ylamino)ethyl]benzene-1,2-diol, monohydrochloride; MW = 248 g/mol; 1 mg/kg ≈ 4 µmol/kg) and propranolol (1-naphthalen-1-yloxy-3-(propan-2-ylamino)propan-2-ol, monohydrochloride; MW = 296 g/mol; 1 mg/kg ≈ 3.4 µmol/kg) were obtained from Millipore Sigma (St. Louis, MO, USA), and RS25344 (1-(3-nitrophenyl)-3-(pyridin-4-ylmethyl)pyrido[2,3-d]pyrimidine-2,4-dione, monohydrochloride; MW = 412 g/mol; 1 mg/kg ≈ 2.4 µmol/kg) was obtained from Santa Cruz Biotech (Santa Cruz, CA, USA). All drugs were initially dissolved in dimethyl sulfoxide (DMSO), subsequently diluted with phosphate-buffered saline (PBS) at a pH of 7.4, containing final concentrations of 5% DMSO and 5% Cremophor EL (Millipore Sigma, St. Louis, MO), and were applied by intraperitoneal (i.p.) injection (100 µL per 20 g body weight). The D-glucose was obtained from Fisher Chemical (Waltham, MA, USA), and insulin (Humulin R U-100) from Lilly USA (Indianapolis, IN, USA).

### 4.2. Animals

Wild-type C57BL/6 mice were bred in-house using founders obtained from Charles River Laboratories (Wilmington, MA, USA). All mice were group-housed, four mice per cage, with *ad libitum* access to food and water, and were maintained in a temperature-controlled (22–23 °C) vivarium with a 12-h light/dark cycle. Adult mice ≥10 weeks of age, ≥18 g in body weight, and of either sex were used for experimentation by equally and randomly dividing the cage littermates into experimental groups. Experimenters were blinded to the identity of the injected drugs until the data acquisition and analyses were completed. Unless indicated otherwise, mice were routinely deprived of food for 5 h prior to experimentation, while having free access to water. For euthanasia, animals were injected i.p. with EUTHASOL^®^ Euthanasia Solution (Patterson Veterinary, Greeley, CO, USA), followed by cervical dislocation. All experiments and procedures were conducted in accordance with the guidelines described in the Guide for the Care and Use of Laboratory Animals (National Institutes of Health, Bethesda, MD, USA) and were approved by the University of South Alabama Institutional Animal Care and Use Committee.

### 4.3. Measurement of Blood Glucose Levels and Insulin Tolerance Tests (ITT)

Blood glucose levels were measured using a Freestyle Lite glucometer (Abbott, Mississauga, ON, Canada) on blood drawn via tail-vein pricks/lancing. These types of point-of-care glucometers are calibrated for use in humans and, while widely used to assess blood glucose levels in animals, may generate somewhat elevated blood glucose readings in mouse whole blood, compared to the glucose levels measured in plasma with biochemical assays [68,69]. For the insulin tolerance tests (ITT), mice were administered insulin (0.625 U/Kg of Humulin R U-100; Lilly USA, Indianapolis, IN, USA) diluted in phosphate-buffered saline (PBS) by intraperitoneal (i.p.) injection, followed by the measurement of blood glucose levels. PBS was used as the solvent control for insulin injections.

### 4.4. Measurement of Core Body Temperature, Isoflurane Anesthesia, and the External Warming of Cages

Core body temperature was measured using a thermocouple thermometer (MicroTherma 2T) with a mouse rectal probe (RET-3), both from Braintree Scientific (Braintree, MA, USA), following the manufacturer’s instructions. For some experimental sets, mice were anesthetized by placing the animals in an anesthesia chamber, ventilated with 3% isoflurane in 100% oxygen at a flow rate of 1.5 L/min. Anesthesia was confirmed by the absence of the toe-pinch reflex. For a portion of the experiments, mouse cages were maintained at 34 °C by external warming of the cages using an electric heater.

### 4.5. ELISA and cAMP-Enzyme Immunoassay (EIA)

To measure serum insulin levels, postprandial mice that had been deprived of food for 5 h were injected with PDE4 inhibitors or solvent control for the indicated time points (15 min, 30 min, and 60 min), and blood samples were then drawn via cheek (submandibular vein) bleed under isoflurane anesthesia and collected in serum-separator tubes. Insulin in the resulting serum samples was determined using a Mouse Insulin ELISA kit from Crystal Chem (Elk Grove Village, IL, USA).

To measure cAMP levels, tissues were rapidly extracted from euthanized animals, flash-frozen in liquid nitrogen, and stored at −80 °C until processing. Tissues were ground into a powder under liquid nitrogen and then suspended in 1 ml of an ice-cold solution comprising 95% ethanol and 5% trichloroacetic acid (TCA). After a 30-min incubation on ice, samples were centrifuged at 12,000× *g* for 10 min. The centrifugation pellet was dissolved in 1 ml of 1 N NaOH and was used to determine protein content. The cAMP-containing supernatant was collected, dried in a SpeedVac concentrator, and subsequently suspended in PBS; the cAMP concentration in these samples was then determined by enzyme immunoassay (EIA) using a kit from Cayman Chemicals (Ann Arbor, MI, USA).

### 4.6. Measurement of Tissue Glycogen Content

Liver and skeletal muscle (gastrocnemius) were rapidly extracted from euthanized animals, flash-frozen in liquid nitrogen, and stored at −80 °C until analysis. To measure the tissue glycogen content, pre-weighed aliquots of frozen tissue were homogenized in 1 ml of ice-cold buffer, comprising 25 mM sodium citrate (pH 4.2) and 60 mM sodium fluoride, using a Dounce glass homogenizer. Samples were then centrifuged at 14,000× *g* for 5 min and the amount of glycogen in the cleared supernatants was assessed via the enzymatic release of glucose, compared to glycogen reference standards, using the EnzyChrom Glycogen Assay Kit (BioAssay Systems; Hayward, CA; E2GN-100), following the manufacturer’s instructions. Tissue glycogen content was calculated as the difference between the total glucose and the free glucose (glucose measured in the absence of glycogen phosphorylase in the assay) in each sample.

### 4.7. Measurement of [^3^H]-2-Deoxy-Glucose Uptake

Mice were deprived of food for 5 h and were then injected with the PDE4 inhibitor roflumilast (5 mg/kg, i.p.) or solvent control. Fifteen minutes later, the mice were injected via the lateral tail vein with 10 µCi of [1,2-^3^H(N)]-deoxy-D-glucose (PerkinElmer, Boston, MA, USA), suspended in a total volume of 100 µL sterile PBS. Thirty minutes later, the mice were euthanized, their tissues extracted, weighed, and heated at 95 °C for 10 min in 1 mL deionized water per ~200 mg tissue. Tissue samples were then homogenized by sonication and centrifuged for 10 min at 12,000 rpm. To separate free, non-phosphorylated [^3^H]-2-deoxyglucose from the phosphorylated [^3^H]-2-deoxyglucose-6-phosphate that reflects cellular uptake, tissue supernatants were diluted to a total volume of 1 mL with water and then passed through columns loaded with 1 ml of anion exchange resin (20% slurry of Bio-Rad AG^®^ 1-X8, #140-1444). After washing the columns twice with 1 mL of deionized water, the [^3^H]-2-deoxyglucose-6-phosphate was eluted from the columns using 2 × 1 ml of 1.5 M NaCl, and the radioactivity in the eluates was then quantified using a liquid scintillation analyzer (PerkinElmer Tri-Carb^®^ 4810 TR). Total radioactivity was corrected for tissue weight and was expressed as pmol of tracer per wet weight of tissue.

### 4.8. Measurement of cAMP-PDE Activity

Tissues were extracted from mice, flash-frozen in liquid nitrogen, and stored at −80 °C until processing. Tissues were then homogenized in a buffer containing 20 mM HEPES (N-2-hydroxyethylpiperazine-N’-2-ethanesulfonic acid) (pH 7.4), 1 mM EDTA (ethylenediaminetetraacetic acid), 0.2 mM EGTA (ethylene glycol-bis(β-aminoethyl ether)-N,N,N′,N′-tetra-acetic acid), 150 mM NaCl, 20% w/v sucrose, HaltTM Protease & Phosphatase Inhibitor Cocktail (Thermo Scientific, Rockford, IL), and 1% Triton X-100, using a Dounce glass homogenizer. After a 30-min rotation at 4 °C, cell debris was pelleted by centrifugation at 20,000× *g* and 4 °C for 10 min, and soluble extracts were then subjected to cAMP-PDE activity assays, as described previously [31]. In brief, the samples were assayed in a reaction mixture of 200 μl containing 40 mM Tris-HCl (pH 7.4), 10 mM MgCl_2_, 1.34 mM β-mercaptoethanol, 1 μM cAMP, and 0.1 μCi [^3^H]cAMP (Perkin Elmer, Waltham, MA, USA) for 10 min at 37 °C, followed by heat inactivation in a boiling-water bath for 1 min. The PDE reaction product, 5′-AMP, was then hydrolyzed by the incubation of the assay mixture with 50 μg of *Crotalus atrox* snake venom (Sigma-Aldrich, St. Louis, MO, USA) for 20 min at 37 °C; the resulting adenosine was afterward separated by anion exchange chromatography on 1 ml of AG1-X8 resin (Bio-Rad Laboratories, Hercules, CA, USA) and quantitated by scintillation counting. PDE4 activity was defined as the fraction of total cAMP-PDE activity inhibited by 10 μM of the archetypal PDE4 inhibitor rolipram compared to solvent control (final concentration of 1% DMSO in the assay reaction).

### 4.9. Measurement of Locomotion Using SmartCageTM Technology

After intraperitoneal injection with the indicated drugs, mice were immediately placed in a new cage with *ad libitum* access to food and water, while locomotion was assessed using the SmartCageTM system (AfaSci Research Laboratories, Redwood City, CA, USA) which tracks the animal’s position and movement in the cage, based on the animal’s disruption of an array of infrared light beams. The travel distance in cm per 5 min time interval was used as a readout of locomotor activity and was plotted over time from the moment the mouse was placed in the cage.

### 4.10. Data and Statistical Analysis

All data are expressed as the mean ± SEM and *n* numbers indicate the number of individual animals assessed. The GraphPad Prism 8.3 software (GraphPad Software, Inc., San Diego, CA, USA) was used to perform statistical analyses. The Mann–Whitney test, with a 95% confidence interval, was used to compare two treatment groups, while the Kruskal–Wallis test, followed by Dunn’s post hoc test, was used to determine the differences between more than two treatment groups. Time courses were analyzed using a two-way ANOVA with Sidak’s post hoc test. Statistical differences are indicated as # (not significant; *p* > 0.05), * (*p* < 0.05), ** (*p* < 0.01), and *** (*p* < 0.001).

## Data Availability

Not applicable.

## Abbreviations

COPD, chronic obstructive pulmonary disease; cAMP, 3′,5′-cyclic adenosine monophosphate; DMSO, dimethyl sulfoxide; EIA, enzyme immunoassay; GLUT4, glucose transporter Type 4; o.g., oral gavage; i.p., intraperitoneal; i.v., intravenous; ITT, insulin tolerance test; PBS, phosphate-buffered saline; PDE, cyclic nucleotide phosphodiesterase; PDE4, cAMP phosphodiesterase 4; PKA, protein kinase A; TCA, trichloroacetic acid.

## References

[1] Rogne M., Taskén K. (2014). Compartmentalization of cAMP signaling in adipogenesis, lipogenesis, and lipolysis. Horm. Metab. Res..

[2] Yang H., Yang L. (2016). Targeting cAMP/PKA pathway for glycemic control and type 2 diabetes therapy. J. Mol. Endocrinol..

[3] Bouchez C., Devin A. (2019). Mitochondrial Biogenesis and Mitochondrial Reactive Oxygen Species (ROS): A Complex Relationship Regulated by the cAMP/PKA Signaling Pathway. Cells.

[4] Gerich J.E. (1993). Control of glycaemia. Baillières Clin. Endocrinol. Metab..

[5] Hers H. (1990). Mechanisms of blood glucose homeostasis. J. Inherit. Metab. Dis..

[6] Dimitriadis G., Maratou E., Kountouri A., Board M., Lambadiari V. (2021). Regulation of Postabsorptive and Postprandial Glucose Metabolism by Insulin-Dependent and Insulin-Independent Mechanisms: An Integrative Approach. Nutrients.

[7] Furman B., Ong W.K., Pyne N.J. (2010). Cyclic AMP signaling in pancreatic islets. Adv. Exp. Med. Biol..

[8] Petersen M.C., Vatner D.F., Shulman G.I. (2017). Regulation of hepatic glucose metabolism in health and disease. Nat. Rev. Endocrinol..

[9] Baillie G.S., Tejeda G.S., Kelly M.P. (2019). Therapeutic targeting of 3′,5′-cyclic nucleotide phosphodiesterases: Inhibition and beyond. Nat. Rev. Drug. Discov..

[10] Conti M., Beavo J. (2007). Biochemistry and physiology of cyclic nucleotide phosphodiesterases: Essential components in cyclic nucleotide signaling. Annu. Rev. Biochem..

[11] Richter W., Jin S.L., Conti M. (2005). Splice variants of the cyclic nucleotide phosphodiesterase PDE4D are differentially expressed and regulated in rat tissue. Biochem. J..

[12] Conti M., Richter W., Mehats C., Livera G., Park J.Y., Jin C. (2003). Cyclic AMP-specific PDE4 phosphodiesterases as critical components of cyclic AMP signaling. J. Biol. Chem..

[13] Paes D., Schepers M., Rombaut B., van den Hove D., Vanmierlo T., Prickaerts J. (2021). The Molecular Biology of Phosphodiesterase 4 Enzymes as Pharmacological Targets: An Interplay of Isoforms, Conformational States, and Inhibitors. Pharmacol. Rev..

[14] Peng T., Qi B., He J., Ke H., Shi J. (2020). Advances in the Development of Phosphodiesterase-4 Inhibitors. J. Med. Chem..

[15] Li H., Zuo J., Tang W. (2018). Phosphodiesterase-4 Inhibitors for the Treatment of Inflammatory Diseases. Front Pharm..

[16] Richter W., Menniti F.S., Zhang H.T., Conti M. (2013). PDE4 as a target for cognition enhancement. Expert Opin. Ther. Targets.

[17] Jin S.L., Ding S.L., Lin S.C. (2012). Phosphodiesterase 4 and its inhibitors in inflammatory diseases. Chang. Gung. Med. J..

[18] Houslay M.D., Baillie G.S., Maurice D.H. (2007). cAMP-Specific phosphodiesterase-4 enzymes in the cardiovascular system: A molecular toolbox for generating compartmentalized cAMP signaling. Circ. Res..

[19] Houslay M.D., Schafer P., Zhang K.Y. (2005). Keynote review: Phosphodiesterase-4 as a therapeutic target. Drug Discov. Today.

[20] Zhang K.Y., Ibrahim P.N., Gillette S., Bollag G. (2005). Phosphodiesterase-4 as a potential drug target. Expert Opin. Targets.

[21] Wu C., Rajagopalan S. (2016). Phosphodiesterase-4 inhibition as a therapeutic strategy for metabolic disorders. Obes. Rev..

[22] Park S.J., Ahmad F., Philp A., Baar K., Williams T., Luo H., Ke H., Rehmann H., Taussig R., Brown A.L. (2012). Resveratrol ameliorates aging-related metabolic phenotypes by inhibiting cAMP phosphodiesterases. Cell.

[23] Muo I.M., MacDonald S.D., Madan R., Park S.J., Gharib A.M., Martinez P.E., Walter M.F., Yang S.B., Rodante J.A., Courville A.B. (2019). Early effects of roflumilast on insulin sensitivity in adults with prediabetes and overweight/obesity involve age-associated fat mass loss-results of an exploratory study. Diabetes Metab. Syndr. Obes..

[24] Wouters E.F., Bredenbröker D., Teichmann P., Brose M., Rabe K.F., Fabbri L.M., Göke B. (2012). Effect of the phosphodiesterase 4 inhibitor roflumilast on glucose metabolism in patients with treatment-naive, newly diagnosed type 2 diabetes mellitus. J. Clin. Endocrinol. Metab..

[25] Ferguson L.D., Cathcart S., Rimmer D., Semple G., Brooksbank K., Paterson C., Brown R., Harvie J., Gao X., Radjenovic A. (2021). Effect of the phosphodiesterase 4 inhibitor apremilast on cardiometabolic outcomes in psoriatic disease—Results of the Immune Metabolic Associations in Psoriatic Arthritis study. Rheumatology.

[26] Mazzilli S., Lanna C., Chiaramonte C., Cesaroni G.M., Zangrilli A., Palumbo V., Cosio T., Dattola A., Gaziano R., Galluzzo M. (2020). Real life experience of apremilast in psoriasis and arthritis psoriatic patients: Preliminary results on metabolic biomarkers. J. Derm..

[27] Jensterle M., Salamun V., Kocjan T., Vrtacnik Bokal E., Janez A. (2015). Short term monotherapy with GLP-1 receptor agonist liraglutide or PDE 4 inhibitor roflumilast is superior to metformin in weight loss in obese PCOS women: A pilot randomized study. J. Ovarian Res..

[28] Mollmann J., Kahles F., Lebherz C., Kappel B., Baeck C., Tacke F., Werner C., Federici M., Marx N., Lehrke M. (2017). The PDE4 inhibitor roflumilast reduces weight gain by increasing energy expenditure and leads to improved glucose metabolism. Diabetes Obes. Metab..

[29] Vollert S., Kaessner N., Heuser A., Hanauer G., Dieckmann A., Knaack D., Kley H.P., Beume R., Weiss-Haljiti C. (2012). The glucose-lowering effects of the PDE4 inhibitors roflumilast and roflumilast-N-oxide in db/db mice. Diabetologia.

[30] Aragon I.V., Boyd A., Abou Saleh L., Rich J., McDonough W., Koloteva A., Richter W. (2021). Inhibition of cAMP-phosphodiesterase 4 (PDE4) potentiates the anesthetic effects of Isoflurane in mice. Biochem. Pharmacol..

[31] Boyd A., Aragon I.V., Abou Saleh L., Southers D., Richter W. (2021). The cAMP-phosphodiesterase 4 (PDE4) controls β-adrenoceptor- and CFTR-dependent saliva secretion in mice. Biochem. J..

[32] McDonough W., Aragon I.V., Rich J., Murphy J.M., Abou Saleh L., Boyd A., Koloteva A., Richter W. (2020). PAN-selective inhibition of cAMP-phosphodiesterase 4 (PDE4) induces gastroparesis in mice. FASEB J..

[33] McDonough W., Rich J., Aragon I.V., Abou Saleh L., Boyd A., Richter A., Koloteva A., Richter W. (2020). Inhibition of type 4 cAMP-phosphodiesterases (PDE4s) in mice induces hypothermia via effects on behavioral and central autonomous thermoregulation. Biochem. Pharmacol..

[34] Boyd A., Aragon I.V., Rich J., McDonough W., Oditt M., Irelan D., Fiedler E., Abou Saleh L., Richter W. (2021). Assessment of PDE4 Inhibitor-Induced Hypothermia as a Correlate of Nausea in Mice. Biology.

[35] Lenhardt R. (2018). Body temperature regulation and anesthesia. Handb. Clin. Neurol..

[36] Doyle M.E., Egan J.M. (2003). Pharmacological Agents That Directly Modulate Insulin Secretion. Pharmacol. Rev..

[37] Fagerholm V., Haaparanta M., Scheinin M. (2011). α2-Adrenoceptor Regulation of Blood Glucose Homeostasis. Basic Clin. Pharmacol. Toxicol..

[38] Ito K., Dezaki K., Yoshida M., Yamada H., Miura R., Rita R.S., Ookawara S., Tabei K., Kawakami M., Hara K. (2017). Endogenous α2A-Adrenoceptor–Operated Sympathoadrenergic Tones Attenuate Insulin Secretion via cAMP/TRPM2 Signaling. Diabetes.

[39] Xin J.Z., Wu J.M., Hu G.M., Gu H.J., Feng Y.N., Wang S.X., Cong W.W., Li M.Z., Xu W.L., Song Y. (2020). α 1-AR overactivation induces cardiac inflammation through NLRP3 inflammasome activation. Acta Pharmacol. Sin..

[40] Hamamdzic D., Duzic E., Sherlock J.D., Lanier S.M. (1995). Regulation of alpha 2-adrenergic receptor expression and signaling in pancreatic beta-cells. Am. J. Physiol..

[41] Bruss M.D., Richter W., Horner K., Jin S.L., Conti M. (2008). Critical role of PDE4D in beta2-adrenoceptor-dependent cAMP signaling in mouse embryonic fibroblasts. J Biol. Chem..

[42] Leroy J., Abi-Gerges A., Nikolaev V.O., Richter W., Lechêne P., Mazet J.L., Conti M., Fischmeister R., Vandecasteele G. (2008). Spatiotemporal dynamics of beta-adrenergic cAMP signals and L-type Ca2+ channel regulation in adult rat ventricular myocytes: Role of phosphodiesterases. Circ. Res..

[43] Rochais F., Abi-Gerges A., Horner K., Lefebvre F., Cooper D.M., Conti M., Fischmeister R., Vandecasteele G. (2006). A specific pattern of phosphodiesterases controls the cAMP signals generated by different Gs-coupled receptors in adult rat ventricular myocytes. Circ Res.

[44] Baillie G.S., Sood A., McPhee I., Gall I., Perry S.J., Lefkowitz R.J., Houslay M.D. (2003). beta-Arrestin-mediated PDE4 cAMP phosphodiesterase recruitment regulates beta-adrenoceptor switching from Gs to Gi. Proc. Natl. Acad. Sci. USA.

[45] Pratt E.P.S., Harvey K.E., Salyer A.E., Hockerman G.H. (2019). Regulation of cAMP accumulation and activity by distinct phosphodiesterase subtypes in INS-1 cells and human pancreatic β-cells. PLoS ONE.

[46] Furman B., Pyne N. (2006). Modulation of cyclic nucleotides and cyclic nucleotide phosphodiesterases in pancreatic islet beta-cells and intestinal L-cells as targets for treating diabetes mellitus. Curr. Opin. Investig. Drugs.

[47] Pyne N.J., Furman B.L. (2003). Cyclic nucleotide phosphodiesterases in pancreatic islets. Diabetologia.

[48] Mutel E., Gautier-Stein A., Abdul-Wahed A., Amigó-Correig M., Zitoun C., Stefanutti A., Houberdon I., Tourette J.A., Mithieux G., Rajas F. (2011). Control of blood glucose in the absence of hepatic glucose production during prolonged fasting in mice: Induction of renal and intestinal gluconeogenesis by glucagon. Diabetes.

[49] Miller R.A., Birnbaum M.J. (2016). Glucagon: Acute actions on hepatic metabolism. Diabetologia.

[50] Imahashi K., Yoshioka J., Yamakita T., Yamano S., Kusuoka H., Nishimura T. (2001). Type IV Phosphodiesterase Inhibitor Suppresses Insulin-Dependent Myocardial Glucose Uptake. Clin. Exp. Pharmacol. Physiol..

[51] Ishikawa M., Hosoi R., Kobayashi K., Nishimura T., Inoue O. (2002). Rolipram depresses [^3^H]2-deoxyglucose uptake in mouse brain and heart in vivo. Eur. J. Nucl. Med. Mol. Imaging.

[52] Ngala R.A., O’Dowd J.F., Stocker C.J., Cawthorne M.A., Arch J.R.S. (2013). β2-adrenoceptor agonists can both stimulate and inhibit glucose uptake in mouse soleus muscle through ligand-directed signalling. Naunyn-Schmiedeberg’s Arch. Pharmacol..

[53] Nevzorova J., Evans B.A., Bengtsson T., Summers R.J. (2006). Multiple signalling pathways involved inβ2-adrenoceptor-mediated glucose uptake in rat skeletal muscle cells. Br. J. Pharmacol..

[54] Potter D.E., Ellis S. (1975). Isoproterenol- and epinephrine-induced changes in blood glucose and tissue glycogen levels in normal and diabetic rats: The influence of alteration in endogenous insulin levels and state of nourishment. J. Pharm. Exp..

[55] Piper R.C., James D.E., Slot J.W., Puri C., Lawrence J.C. (1993). GLUT4 phosphorylation and inhibition of glucose transport by dibutyryl cAMP. J. Biol. Chem..

[56] Lawrence J.C., Piper R.C., Robinson L.J., James D.E. (1992). GLUT4 facilitates insulin stimulation and cAMP-mediated inhibition of glucose transport. Proc. Natl. Acad. Sci. USA.

[57] Niu W., Bilan P.J., Hayashi M., Da Y., Yao Z. (2007). Insulin sensitivity and inhibition by forskolin, dipyridamole and pentobarbital of glucose transport in three L6 muscle cell lines. Sci. China Ser. C Life Sci..

[58] Tan V.P., Miyamoto S. (2015). HK2/hexokinase-II integrates glycolysis and autophagy to confer cellular protection. Autophagy.

[59] Roberts D.J., Miyamoto S. (2015). Hexokinase II integrates energy metabolism and cellular protection: Akting on mitochondria and TORCing to autophagy. Cell Death Differ..

[60] Wilson J.E. (2003). Isozymes of mammalian hexokinase: Structure, subcellular localization and metabolic function. J. Exp. Biol..

[61] Ritov V.B., Kelley D.E. (2001). Hexokinase Isozyme Distribution in Human Skeletal Muscle. Diabetes.

[62] Brás N.F., Fernandes P.A., Ramos M.J., Schwartz S.D. (2017). Mechanistic Insights on Human Phosphoglucomutase Revealed by Transition Path Sampling and Molecular Dynamics Calculations. Chem.–A Eur. J..

[63] Fueger P.T. (2005). GLUCOSE PHOSPHORYLATION AS A BARRIER TO MUSCLE GLUCOSE UPTAKE. Clin. Exp. Pharmacol. Physiol..

[64] Jeukendrup A.E. (2006). Regulation of Fat Metabolism in Skeletal Muscle. Ann. N. Y. Acad. Sci..

[65] Reitman M.L. (2018). Of mice and men—Environmental temperature, body temperature, and treatment of obesity. FEBS Lett..

[66] Demetrius L. (2005). Of mice and men. EMBO Rep..

[67] Jensterle M., Kocjan T., Janez A. (2014). Phosphodiesterase 4 Inhibition as a Potential New Therapeutic Target in Obese Women With Polycystic Ovary Syndrome. J. Clin. Endocrinol. Metab..

[68] Morley L.A., Gomez T.H., Goldman J.L., Flores R., Robinson M.A. (2018). Accuracy of 5 Point-of-Care Glucometers in C57BL/6J Mice. JAALAS.

[69] Togashi Y., Shirakawa J., Okuyama T., Yamazaki S., Kyohara M., Miyazawa A., Suzuki T., Hamada M., Terauchi Y. (2016). Evaluation of the appropriateness of using glucometers for measuring the blood glucose levels in mice. Sci. Rep..

